# Adolescents Conceived through Donor Insemination in Mother‐Headed Families: A Qualitative Study of Motivations and Experiences of Contacting and Meeting Same‐donor Offspring

**DOI:** 10.1111/chso.12158

**Published:** 2016-04-14

**Authors:** Sherina Persaud, Tabitha Freeman, Vasanti Jadva, Jenna Slutsky, Wendy Kramer, Miriam Steele, Howard Steele, Susan Golombok

**Affiliations:** ^1^ Center for Attachment ResearchDepartment of Clinical PsychologyThe New School for Social ResearchNew YorkNYUSA; ^2^ Centre for Family ResearchDepartment of PsychologyUniversity of CambridgeCambridgeUK; ^3^ Donor Sibling RegistryNederlandCOUSA

**Keywords:** donor conception, donor insemination, donor siblings, same‐donor offspring, sperm donation

## Abstract

This study interviewed adolescents conceived using sperm donation to examine their experiences of contacting and meeting ‘same‐donor offspring’ (i.e. donor‐conceived offspring raised in different families who share the same donor), their motivations for this contact, and how they make meaning of these relationships. This in‐depth qualitative study involved semi‐structured interviews with 23 young people aged 12–19 years (mean = 14 years). Interviewees were motivated by curiosity about their biological relations and by wanting to extend their family. Contact with same‐donor offspring was described as being either normal/neutral or as a unique experience that was integrated into their identity. This study highlights the importance of contact between same donor offspring, particularly during adolescence, a developmental stage associated with identity formation. The findings have important policy implications as they suggest that donor‐conceived individuals may benefit from contact with others conceived using the same donor prior to the age of 18 years.

## Introduction

Advances in assisted reproductive technologies have helped many individuals to conceive children through the use of donated sperm and eggs. As more families begin to utilise gamete donation as a means to achieve parenthood, the possibility of connecting to other ‘same‐donor offspring’ (i.e. offspring conceived using the same donor growing up in different families; Freeman, Jadva, Slutsky, 2016) has become increasingly salient. The number of births from donor conception is not systematically regulated. Although the American Society for Reproductive Medicine has recommended guidelines limiting each sperm donor to 25 live births per population area of 850 000, there is no external monitoring or enforcement by law (Spar, 2006). Therefore, the information available to families about the potential number of same‐donor offspring is limited.

The relationships formed between these families of biologically related siblings create unique family systems that are facilitated by registries and changes in anonymity regulations. For example in the USA, some clinics offer either anonymous or open‐identity donors (i.e. where the donor's identity is released to the child on reaching a specific age). In Sweden and parts of Australia, New Zealand and throughout Europe, policies have been implemented that require sperm donors to disclose their identity (Blyth and Frith, 2009). In the United Kingdom, the amended Human Fertility and Embryology Act (1990) permits children conceived after 2005 to access identifying information about their donor and any same‐donor offspring (provided these ‘genetic siblings’ consent to such disclosure) after age 18 years, with non‐identifying information being available from age 16 years. However, it is possible for individuals conceived using gamete donation to seek out their donor connections before this age by utilising online registries such as the Donor Sibling Registry, an international registry based in the USA (www.donorsiblingregistry.com). Other mechanisms for facilitating donor connections are available in other countries and these operate in different ways (Crawshaw and others, 2015). It is therefore possible for donor‐conceived offspring to connect with their donor relations during childhood and to grow up knowing and, in some cases, to have ongoing contact with their donor siblings and/or donor.

Research examining the experiences of donor‐conceived individuals who search for and contact their donor relations has so far been conducted using survey methodology. Studies of parents have found that the primary motivation for searching for their child's donor siblings was ‘curiosity’ (Freeman and others, 2009; Hertz and Mattes, 2011), and the main reason for searching for the donor was to ‘enhance their child's sense of identity’ (Freeman and others, 2009). Single mothers and lesbian couples were more likely to be searching for donor siblings than were heterosexual couples (Freeman and others, 2009) motivated by a desire to create family for the child and to obtain more information about the donor (Hertz and Mattes, 2011; Scheib and Ruby, 2008). Parents reported that their children's relationships with their donor siblings ranged from a distant relative to much closer relationships (Hertz and Mattes, 2011).

Far fewer studies have obtained information directly from donor‐conceived individuals about their experiences of searching for and contacting same donor offspring. In a review of 19 articles that have been published between the years 2000 and 2011 on the topic of donor‐conceived offspring's views of their donor conception, Blyth and colleagues found that several of these studies reported on participants’ interest in other same‐donor offspring (Blyth and others, 2012). Just four of these studies addressed experiences of contacting same‐donor offspring, and two of these examined the quality of these experiences (Blyth, 2012b; Jadva and others, 2010). Studies have mostly used surveys or email interviews and have found that most donor‐conceived individuals report positive experiences of making contact with their donor siblings (Jadva and others, 2010) with donor‐conceived adults reporting that contact with their donor siblings has enabled them to gain more information about their genetic background and has contributed to redefining their identity (Blyth, 2012a).

Differences have been found between offspring from heterosexual couple families and those from lesbian couple families, with donor‐conceived individuals with heterosexual parents being less likely to discuss searching for their donor connections with their father when compared with children with lesbian parents who were more likely to inform their non‐genetically related parent (Jadva and others, 2010; Beeson and others, 2011). Reports from lesbian mothers of 17‐year olds demonstrated that they were more dissatisfied with unknown donors than with open‐identity donors, and that this was mainly related to access to the donor and concerns about custody (Gartrell and others, 2014). The development of complex familial and kinship relationships has been highlighted in a qualitative study of 11 donor‐conceived individuals ages 19–29 years with known donors raised by lesbian parents in which participants described a range of experiences from viewing their donor as solely a donor to having involved relationships with the donor (Goldberg and Allen, 2013). This study also emphasised the importance of the young adult developmental period as one in which the relationship with their donor shifts as they take on more responsibility for managing this relationship.

That donor‐conceived individuals are connecting with their donor relations prior to adulthood is of significance and raises questions about how these relationships contribute to the children's development at different stages of their lives. Adolescence, for instance is a critical time for identity formation (Erikson, 1959). From early to late adolescence, children are negotiating an individuation process from their parents, which ultimately results in separation from them in terms of their identity (Collins and Laursen, 2004; Collins and Steinberg, 2006). This separation enables the formation of a unique identity, a goal of adolescence, which can result in conflict in relationships with parents (Smetana and others, 2006). As the introduction of identity release policies start to allow children to have access to information about same‐donor offspring and their donor, studying how these relationships are formed and experienced during this developmental stage becomes increasingly relevant. Due to the importance of identity formation in adolescence, it is important to understand better the process by which adolescents integrate donor relations into their personal narratives. Specifically, how adolescents respond to their donor conception, how they make meaning of their relationships with their donor relations, and how this affects pre‐existing familial relationships. This understanding can inform policy decisions about the age at which children should have identifying information about their donor relations.

This study is the first to conduct in‐depth, face‐to‐face interviews with young people about their experiences of contacting and meeting same‐donor offspring and aimed to increase understanding of how they make meaning of their relationships with genetically related offspring. In particular, themes will be presented describing how their connection to same‐donor offspring is experienced and the social and psychological roles that they desire from these relationships. The study focuses on donor‐conceived children of lesbian couples and single mothers as children in families without a father are more likely to be told of their donor conception at an earlier age, and are more open with their parents about their search for donor connections (Beeson and others, 2011; Jadva and others, 2010).

## Materials and methods

In‐depth face‐to‐face interviews were conducted with 23 young people conceived via anonymous sperm donation on their experiences of contacting and meeting same‐donor offspring. Parents of donor offspring who had contacted at least one same‐donor offspring and resided in New York, New Jersey, or Connecticut were contacted via email from the Donor Sibling Registry. The Donor Sibling Registry is a website created in 2000 that connects egg, sperm and embryo donor offspring with same‐donor offspring as well as their donors. Members willing to be contacted by the research team were telephoned and an in‐home interview date was scheduled for those who wished to take part. Participants in this study included 16 girls and 7 boys aged 12–19 years (mean = 14 years). A total of 25 participants whose parents had consented to being interviewed were invited to take part in this study. Of these, 23 agreed to be interviewed (92 per cent) and 2 declined.

Semi‐structured interviews lasting approximately 60–90 min were conducted in participants’ homes. The interview inquired about participants’ sense of what it meant to be donor conceived and explored the terminology used to describe same‐donor offspring and donors. The interview also examined adolescents’ experiences of learning about their conception, motivations for searching for same‐donor offspring, experiences of contacting and meeting families sharing the same donor, information known about their donor, and feelings about not knowing their donor. This paper reports on sections of the interview relating to the experiences of meeting and contacting same‐donor offspring as well as feelings regarding donor conception.

Interviews were recorded and transcribed verbatim and analysed using thematic analysis (Braun and Clarke, 2006). Themes were identified on a semantic level and reported as frequencies aimed at reflecting participants’ experiences of contacting and meeting same‐donor offspring and how they make meaning of this experience. As this analysis was conducted inductively, identified themes did not fit into a pre‐existing theory, and instead articulated the nuances of the participants’ experiences. Pseudonyms have been used throughout this paper to protect the confidentiality of participants. Identifying information has also been removed. Written informed consent to participate in the study was obtained from all participants aged over 18 years; those under 18 years gave their assent and their parents gave their written consent for their children to take part. The study was granted ethical approval from the University of Cambridge Psychology Ethics Committee and The New School Institutional Review Board.

### Sample characteristics

Fourteen participants were from families headed by single mothers, 10 of the mothers identified as heterosexual, 2 as bisexual and 2 chose not to specify their sexual orientation. Nine participants were from families headed by mothers in same‐sex couples, most of the mothers identified as lesbian and 1 identified as bisexual. Eight (35 per cent) participants had one sibling (mean age = 11.1 years) residing with them; of these eight siblings, five were conceived with the same donor sperm as the participant, one was adopted and two were conceived using a different donor. With regards to the age at which they had found out about their donor conception, four (18 per cent) participants reported having ‘always known’, nine (39 per cent) ‘could not recall’, nine (39 per cent) found out before the age of 7 and one (4 per cent) found out after the age of 10. Twenty‐two (96 per cent) participants were identified as White and one (4 per cent) as ‘Other, unspecified'. There were two sets of siblings who were raised in the same family who participated in the study.

## Results

### Motivations for contact

Two main themes were identified as motivations for contact with same‐donor offspring: ‘Curiosity about donor and genetic origins’ and ‘forming relationships to extend family’.

### Curiosity about donor and genetic origins

The most common motivating factor for contacting and meeting same‐donor offspring was a sense of curiosity (*n* = 19). Some participants said that they had not been aware of the existence of other same‐donor offspring (*n* = 7) and that they felt excited to learn about the possibility of meeting them:

I didn't know that more people used the sperm from the gu‐same guy… I didn't know that anyone else would buy the sperm from the same guy as us and that I didn't, I didn't even know half‐families existed so when I first met them it was pretty cool. (Anthony)

Most participants (*n* = 10) were driven to contact same‐donor offspring as a means of obtaining more information about their donor. By comparing themselves to their donor siblings, participants were able to identify characteristics that they thought could originate from the donor and provide them with a better understanding of him:

If it's something we, we both have like in common it's like ‘oh well it's probably from like our donor’ or like once um we were like talking, I was talking about like how I'm good at English and we're both good at math, she's not good at English though, I and um it was just kind of like ‘well he was an English teacher and he had a perfect score on his SAT's for math and stuff’ like so like we probably got that from him and um and like he's also really ugly so sometimes we're like ‘I don't know where we came from because we're so beautiful (said in playful tone) but he's like disgusting.’ (Veronica)

Although most participants (*n* = 14) indicated that they were curious to meet same‐donor offspring to better understand their origins, not all expressed a desire to contact or meet their donor. For some (*n* = 6), meeting same donor offspring was seen as transformative, as it provided insight into themselves. One participant described how meeting same‐donor offspring had provided him with the opportunity to explore aspects of himself:

It's been great it was an awesome opportunity. There's nothing, I would do it all the same way again. It's yeah it's been great, I mean it's been awesome to meet them, hang out with them, have this new kind of relative, explore like I mean through meeting them I've gotten to know more about myself you know and uh you know who I am, what the donor's like… I mean it's helped me learn about you know how these uh relationships were gonna work like how it would work if I met my father or how um you know about, taught me about you know brother relationships it's, it's cultivated, given me more inspiration in music and stuff… and that's gonna be three new really good friends and brothers (Gregory)

Indeed, most participants (*n* = 13) stated that knowing others who share half their genetic makeup was important to them because it provided them with a more nuanced sense of identity. For some (*n* = 4), simply the fact of knowing about same‐donor offspring provided this enhanced sense of self and they did not necessarily need to meet their donor siblings to achieve this. For others (*n* = 3), the concept of sharing genes with same‐donor offspring motivated them to make contact, even if they did not feel that the experience was overtly significant:

It's been I wouldn't say life changing but it's definitely something that I will remember ‘cause I know that I now have sisters who also are donor conceived and we have the same DNA and stuff like that, well half the same DNA. (Joanna)

### Forming relationships to extend family

Another motivation given by most participants for contacting and meeting same‐donor offspring was to form a relationship with them (*n* = 15). For some (*n* = 4), this relationship was portrayed as multifaceted in that the donor sibling was experienced as in between a friend and a family member. For three other participants who had no siblings residing with them, finding same‐donor offspring provided them with someone who could fulfil a perceived ‘missing’ role in their family. Participants described how their bond to a particular same‐donor offspring helped to serve such a role:

I'm really just like happy with like just her cause she real–she kind of like filled the place of needing to find like another sister or like someone who's really like me and so I still really, so I do still like see like other siblings but for the most part I really just like spending time with [Donor Sibling Sister] cause I just, I love seeing her. (Veronica)

I thought it was cool, it wasn't like I was kind of envious of not having a dad, um I was more I mean if I was ever envious of any family relationship I kind of wanted a brother or something, which I now have. (Gregory)

For Veronica, being close to just one of her same‐donor offspring gave her a sense of having a sister which was enough for her. Similarly, Gregory describes how knowing about a same‐donor offspring had given him a brother, which he found fulfilling. These donor relations were often described as complex; sometimes experienced as awkward at first but then developing into a closer relationship (*n* = 4). In these cases, connecting with same‐donor offspring was uniformly viewed as enhancing their sense of family, whether the experience was found to be positive, neutral or awkward. Of those participants who were aware of multiple same‐donor offspring, most (*n* = 5) participants developed a close familial relationship with a few and felt relatively distant to others:

I'm best friends with my brother. Like I have two other ones that are girls that like me, um my brother and my two sisters. Like they, we get together sometimes just us ‘cause we're the oldest ones. And we have like a lot of fun together and so we're just like kind of the group. And yeah, the other ones I don't really feel close to. Like they're, I'm aware they're there but I don't like love them like I love those three. (Chelsea)

A third of participants (*n* = 7) expressed that it would be interesting to know about more donor siblings but that it may be difficult to connect with all of them, particularly once a connection had been made to at least one other same‐donor offspring. Unlike siblings who live and grow up together, most participants were introduced to their donor siblings later in life and special trips were required to visit them. Although participants often described their relationship with same‐donor offspring as familial, the relationship was complex as there was an expectation to feel close to their donor siblings despite possible feelings of awkwardness or distance.

### Experience of contacting and meeting same‐donor offspring

Two main themes were identified to describe experiences of contact with same‐donor offspring: ‘normalcy/neutrality’ and a ‘unique experience as part of identity’.

### Normalcy/neutrality of experience

Most participants described having a sense of knowing about their donor conception from an early age, such that it felt like they had always known (*n* = 16). All but two participants articulated that their parent(s) had integrated their donor conception into their lives in such a way that it became a normal part of their identity growing up. This sense of normalcy about their donor conception translated into how they perceived contacting and meeting same‐donor offspring as it provided a foundation from which participants could understand this relationship. While most participants described being excited about the possibility of meeting same‐donor offspring (*n* = 16), some felt indifferent (*n* = 4). In either case, it appeared that adolescents’ feelings of normalcy or indifference about their donor conception was related to them perceiving connecting with same‐donor offspring as being normal.

In one case, a participant said that although she perceived connecting with same‐donor offspring to be normal for her, she was aware of the fact that this might not be perceived as normal to others. Most participants (*n* = 16) described subjectively feeling neutral about the idea of same‐donor offspring; it is when their differences from others are accentuated that they felt a discrepancy.

Most participants (*n* = 13) described feeling that their donor conception was a part of who they were and that fundamentally they did not differ from other families. The majority of participants (*n* = 19) felt comfortable telling others that they had contacted or met same‐donor offspring. While participants could recognise that others may find it ‘different’ to be in contact with donor siblings, most adopted terminology to describe same‐donor offspring in a normative manner. Some participants referred to same‐donor offspring as a ‘half sibling’ (*n* = 9) or ‘donor sibling’ (*n* = 5), whereas others reserved the term ‘brother’ or ‘sister’ for same‐donor offspring to whom they felt particularly close (*n* = 5).

### Unique experience as part of identity

Most participants (*n* = 9) reported that being donor conceived and knowing same‐donor offspring provided them with a special experience that set them aside from others. Sharing this experience with other same‐donor offspring provided some participants with an understanding of their own donor‐conceived identity, as they were able to make meaning of this in the context of a shared experience:

I was so happy about it…, I thought it made me so interesting like I was the most interesting person. I thought it was so cool, like I would tell everyone about it… like I am always open to telling people I think, people are always so interested and I think it really makes people like ‘Oh wow, she's a really interesting person’. And it is really interesting especially now that I have all my donor siblings in the mix ‘cause it doesn't feel as confusing. It doesn't feel like as much of a mystery. (Chelsea)

Other participants (*n* = 4) felt that the experience of contacting and meeting same‐donor offspring gave them a unique part of their narrative of who they are. This contributed to their experience of finding it valuable to connect with same‐donor offspring. Participants expressed an appreciation of the fact that they were able to connect with same‐donor offspring and that they were able to form a relationship that was unlike others. Most noted that it provided them with a ‘quirky story’ that they were pleased to integrate into their lives.

Although being donor‐conceived and seeking same‐donor offspring provided an unusual experience that most participants liked about their life story, being brought up in a single mother or lesbian couple family sometimes produced ambivalent feelings related to not having a father:

…I have a different situation so that's not always easy. There's always people who are like ‘wait, like where's your dad or like what's going on?’ and then there's Father's Day and it's like I don't have that. But it's just, you know, I'm just different. I kinda like that, I guess. (Gina)

Whilst elsewhere in the interview Gina described feeling that she did not need ‘that extra person’ when referring to her donor, here she suggested that a part of her longs for a father. This difficulty appeared to be contextual for Gina in situations in which she was reminded of her status as socially different from others (e.g. during holidays such as Father's Day). Gina concluded that she ultimately liked being different. However, she qualified this statement with ‘I guess’, indicating that there was perhaps some underlying uncertainty about this.

## Discussion

This study highlights the importance of understanding the motivations, experiences and sense of meaning that young people make of contacting and meeting same‐donor offspring. Participants in this study delineated motivations of curiosity and wanting to build familial relationships, and described the experience of meeting same‐donor offspring as a unique one that they had integrated into their sense of normality. In line with other research exploring the experiences and motivations of connecting with same‐donor offspring (Hertz and Mattes, 2011; Jadva and others, 2010), this study also found curiosity and the possibility of having a larger, extended family as motivating factors for donor offspring to contact same‐donor offspring. However, through a close analysis of interviews with these participants, a more nuanced understanding of these motivations, as well as other aspects of their experiences has emerged.

While it is true that most participants described feeling curious about contacting and meeting same‐donor offspring, this curiosity centred on what information they could gain about their donor, and subsequently themselves, from this contact. Through identifying similarities and differences between themselves and same‐donor offspring, participants could obtain a better sense of their genetic background and identity. This desire to know more about their origins is developmentally appropriate in adolescence, as identity formation is at its peak. Questions regarding what makes them unique and also similar to others are a central theme of ego development at this time (Marcia, 1980). This finding calls into question policies that provide identifying information about donor connections at the later age of 18 years.

Our findings are in line with Hertz and Mattes (2011)'s online survey of parents of children conceived through donor insemination that showed the desire to have a larger, extended family to be a motivating factor for contacting same‐donor offspring. In particular, this study found that young people often described same‐donor offspring as fulfilling a relationship that was perceived to be lacking in their lives. Again, this finding appears to be developmentally appropriate as connections to similar others become important in the process of developing an integrated identity in adolescence (Erikson, 1959). However, it is important to note the possible influence of social context in this finding. As it is socially acceptable to form close relationships to those with whom one has a genetic bond, most participants described feeling that they wanted to contact or meet other same‐donor offspring. Once contact was made, however, participants described a range of multifaceted relationships to their donor siblings, ranging from distant, to like a best friend, to like a sibling. Such variation illustrates that although participants cite the desire to extend their family as a motivation for connecting with same‐donor offspring, the subsequent relationship formed with them can be quite different. It is also important to recognise that individuals’ descriptions of relationships do not necessarily reflect their feelings about these relationships, and that their feelings may vary widely.

The participants in this study were all from lesbian couple or single mother families and thus did not have a father in the home. Although a few participants described a sense of lacking a father figure, most articulated that they were generally indifferent about it and this appeared to be a consequence of integrating their family structure and donor conception into their identity from a young age. Certain social situations (e.g. Father's Day) led to feelings of being different to others; however, these differences were not about their donor conception *per se*, but about not having a father. Children in same‐sex couple families form their own interpretations of their social surroundings which can lead to them being selective about whom they discuss their family structure with (Bosisio and Ronfani, 2016) Establishing a basic sense of the differences between oneself and others at an early age enables a better understanding of one's identity during adolescence in a clear and cohesive manner (Marcia, 1980). Acknowledgement of donor conception origins from an early age therefore appears to be an important factor in normalising this experience. Not having a father, while potentially contributing to feelings of being different, does not appear to be associated with feeling uncomfortable with donor conception for participants in our study.

In line with the relevance of identity formation during adolescence, participants described their experience of contacting same‐donor offspring as a unique one that provided them with a sense of being special. Particularly during adolescence, there is a pressure to differentiate the self from others in important and personally meaningful ways (Smetana and others, 2006). It is therefore explicable that participants would assimilate this unique experience into their identity formation and find ways to create a personal narrative inclusive of this experience.

The experiences and motivations of the participants in this study are of relevance to other donor offspring and their parents who are considering connecting with same‐donor offspring as it can provide helpful insight into possible outcomes of this connection. It is clear that some participants were not initially aware of the possibility of other donor‐conceived children sharing their donor, let alone that they could be in contact with them. By making these experiences explicit, families who may not have considered connecting with donor relations can be informed of what these relationships can be like. Likewise, as cultural and policy transitions are leading towards increasing numbers of families who share the same donor seeking contact with each other, empirical studies are important for informing policy‐makers and professionals of the potential outcomes of making these connections.

While this paper provides insight into the experiences of donor‐conceived individuals from single mother and dual parent lesbian households, it does not examine the experiences of donor‐conceived individuals from dual parent heterosexual households. The research currently available on donor‐conceived individuals raised by heterosexual parents is scarce and this may be related to the fact that families from single mother and lesbian couples tend to disclose donor origins to their children more frequently than heterosexual couples (Golombok and others, 2006). In addition, this study examines the experiences of donor‐conceived individuals with anonymous sperm donors. The participants in this study were told of their donor origins at an early age and may have different experiences of both their donor conception and connecting to other same‐donor offspring than if they were told at a later age. Future research could examine how factors such as age of disclosure and type of donor (i.e. identity release, known or anonymous) impact on how donor‐conceived individuals conceptualise their experience of being donor conceived and how they navigate connecting to donor relations.

## Funding

This research was supported by the Wellcome Trust (097857/Z/11/Z).
